# Strategies for the hypothermic preservation of cell sheets of human adipose stem cells

**DOI:** 10.1371/journal.pone.0222597

**Published:** 2019-10-15

**Authors:** Sara Freitas-Ribeiro, Andreia Filipa Carvalho, Marina Costa, Mariana Teixeira Cerqueira, Alexandra Pinto Marques, Rui Luís Reis, Rogério Pedro Pirraco

**Affiliations:** 1 3B's Research Group, I3Bs–Research Institute on Biomaterials, Biodegradables and Biomimetics, University of Minho, Headquarters of the European Institute of Excellence on Tissue Engineering and Regenerative Medicine, Guimarães, Portugal; 2 ICVS/3B’s–PT Government Associate Laboratory, Braga/Guimarães, Portugal; 3 The Discoveries Centre for Regenerative and Precision Medicine, Headquarters at University of Minho, Guimarães, Portugal; EFS, FRANCE

## Abstract

Cell Sheet (CS) Engineering is a regenerative medicine strategy proposed for the treatment of injured or diseased organs and tissues. In fact, several clinical trials are underway using CS-based methodologies. However, the clinical application of such cell-based methodologies poses several challenges related with the preservation of CS structure and function from the fabrication site to the bedside. Pausing cells at hypothermic temperatures has been suggested as a valuable method for short-term cell preservation. In this study, we tested the efficiency of two preservation strategies, one using culture medium supplementation with Rokepie and the other using the preservation solution Hypothermosol, in preserving human adipose stromal/stem cells (hASC) CS-like confluent cultures at 4°C, during 3 and 7 days. Both preservation strategies demonstrated excellent ability to preserve cell function during the first 3 days in hypothermia, as demonstrated by metabolic activity results and assessment of extracellular matrix integrity and differentiation potential. At the end of the 7^th^ day of hypothermic incubation, the decrease in cell metabolic activity was more evident for all conditions. Nonetheless, hASC incubated with Rokepie and Hypothermosol retained a higher metabolic activity and extracellular matrix integrity in comparison with unsupplemented cells. Differentiation results for the later time point showed that supplementation with both Rokepie and Hypothermosol rescued adipogenic differentiation potential but only Rokepie was able to preserve hASC osteogenic potential.

## Introduction

According to the annual report of Organ Procurement and Transplantation Network/The Scientific Registry of Transplant Recipients, in 2017, 115,000 people in the USA alone were waiting for an organ transplant [1] and this number is increasing every year. Unfortunately, the demand largely overcomes the availability, as just 31,768 organs were received in the same year, causing the daily death of around 20 people waiting for an organ [1]. Tissue Engineering and Regenerative Medicine (TERM) strategies are seen as promising approaches to solve the issue of organ shortage [2]. However, limitations of traditional TERM strategies such as low anchorage to the desired site in the case of cell injection [3], strong host reaction in response to the biodegradation of the scaffolds [4], or insufficient delivery of oxygen and nutrients to the bulk of scaffolds, are precluding their widespread clinical application.

A number of scaffold-free approaches have been proposed to surpass the limitations of scaffold use. Of those, one of the most promising is the use of cell sheets [5–7]. This approach allows building completely biologic thick tissues using hyperconfluent cells as extracellular matrix (ECM)-rich building blocks. The ECM is in fact a critical feature of cell sheets since it provides both mechanical and biochemical support and fosters prompt and effective adhesion to tissues. Numerous studies have shown the potential of this approach for the regeneration of a wide range of tissues [8] such as cornea [9], myocardium [10], articular cartilage [11], bone [5] and famously, skin [12]. Given that cell sheets are in fact living tissue-like constructs, the widespread clinical application of cell sheet-based therapies may depend on the development of successful preservation strategies that maintain the structural features and function of cell sheets from the fabrication site to the final destination. This is in fact a major issue in other contexts such as, for e.g., in the case of fabricated skin tissue models [13,14].

Cryopreservation is the gold standard for single cell preservation, and it has also been explored for the preservation of tissues. However, its efficiency on tissues varies depending on the tissue to which it is applied [15] as it exposes cells to extreme conditions that can cause extensive damage [16]. Pausing cells at hypothermic temperatures is a short-term and simplified alternative to cryopreservation [17–20]. This methodology is capable of slowing metabolic activity, protein synthesis, transport systems and cell cycle progression [21,22], and in this way pausing cells in a low energy consumption state. Furthermore, it prevents cell damage from ice nucleation and changes in solute concentration caused by severe temperature changes as the ones experienced by cells during cryopreservation. However, hypothermic preservation is not a method free of deleterious effects to cells and, in the long term, may lead to cell injury and death [23].

Currently, there are a number of hypothermic preservation solutions originally developed for whole organ transplantation such as UW (University of Wisconsin Solution), Celsior and HTK (Histidine-tryptophan-ketoglutarate). Hypothermosol (HTS), on the other hand, is a solution developed to meet the specific requirements of cells and tissues used for regenerative medicine purposes, namely by supporting cell metabolism and inhibiting post-storage necrosis and activation of apoptosis in response to hypothermic temperatures [24]. Recently, Rokepie (RP), a 6-chromanol derivate (SUL-109), has been made available in the market. This molecule is a media supplement that increases the activity of complexes I and IV of the mitochondria, ATP production and minimizes the production of reactive oxygen species (ROS) [20].

In this study, we address the efficiency of two preservation strategies using either RP or HTS, in pausing cell sheet like monolayers of confluent hASCs, at the hypothermic temperature of 4°C, for up to 7 days. At the end of each preservation period, a recovery phase of 24 hours at 37°C was performed and then cell morphology, viability, phenotype, protein expression and ECM integrity were assessed. Furthermore, maintenance of hASC differentiation potential after hypothermic storage towards the adipogenic and osteogenic lineages, commonly undertaken using these progenitor cells, was evaluated.

## Materials and methods

### Isolation of human adipose stromal cells

Human subcutaneous adipose tissue was obtained from liposuction procedures performed at Hospital da Prelada (Porto), after patient’s written informed consent, and in the scope of a collaboration protocol approved by the ethical committees of both institutions for this work (Comissão de Ética do Hospital da Prelada/3B’s Research Group). hASCs were obtained as previously described [6]. Briefly, lipoaspirates were digested with a solution of 0.05% (w/v) collagenase type II (Sigma Aldrich, USA), for 45 minutes at 37°C under agitation, and then centrifuged to obtained stromal vascular fraction (SVF). The obtained SFV was incubated with red blood lysis buffer, centrifuged and the supernatant resuspended in Minimum Essential Medium alpha-modification (α-MEM) (Life Technologies, United Kingdom) supplemented with 10% fetal bovine serum (FBS) and 1% antibiotic/antimycotic. The nucleated cells were counted using a solution of 3% acetic acid (VWR, United Kingdom) and 0.05 wt % methylene blue (Sigma Aldrich, USA) in water, plated in culture flasks (Falcon, United Kingdom) in the same medium as before, expanded in culture with frequent medium changes and passaged after reaching 80% confluence. At the start of the experiments, cells were detached, centrifuged and plated in multiwell polystyrene plates at a density of 15,500 cells/cm^2^. hASCs were then grown for 3 days to stabilize the cultures in monolayers, thus mimicking cell sheets. Cells were used up to passage 4.

### Testing of hypothermic preservation solutions

**Fig 1:**
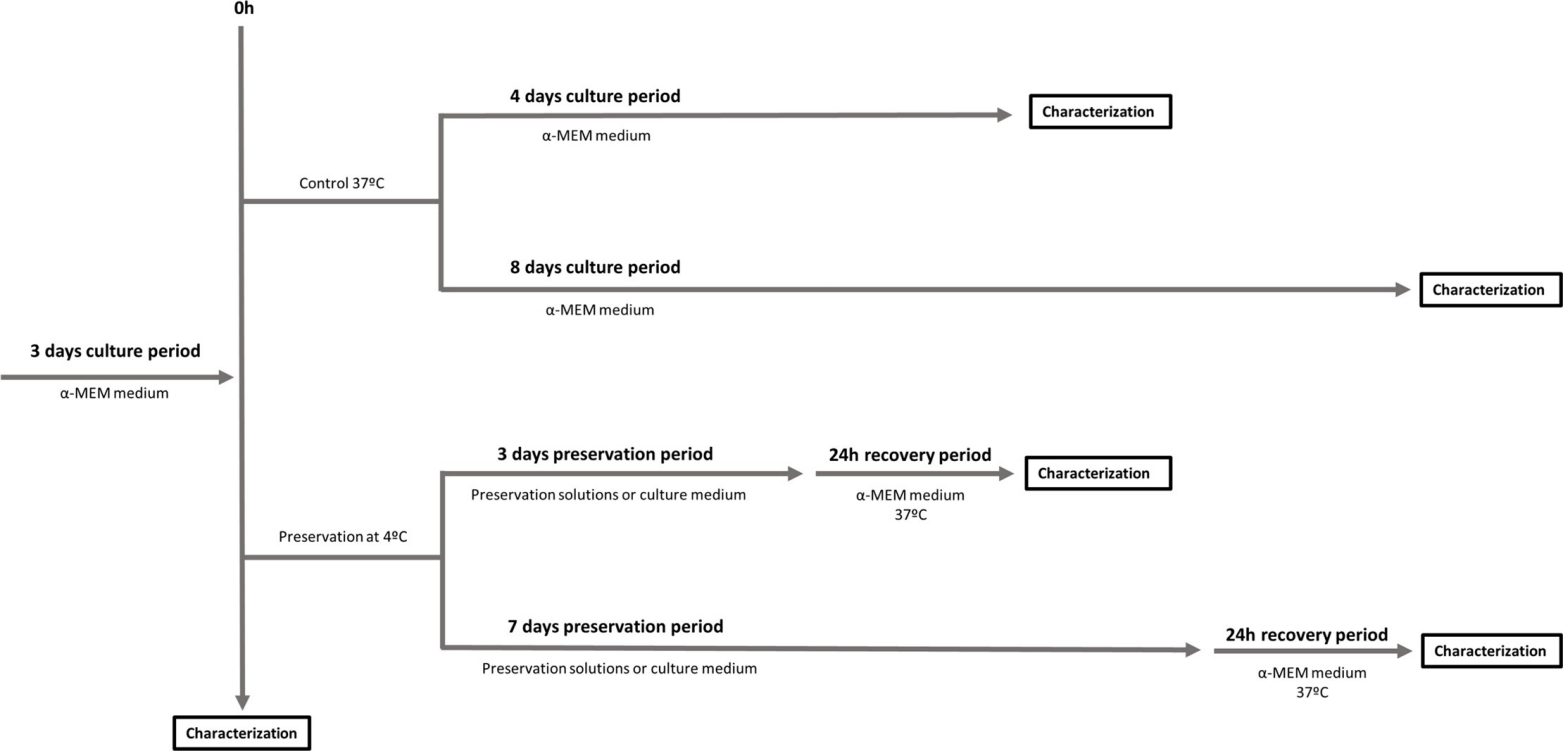
Schematic overview of the overall methodology followed along the work.

Two hypothermic preservation solutions, HTS (Tebu-Bio, France) and RP (Sulfateq, Netherlands) were used. While HTS is a ready to use solution, RP is a media additive. After preliminary testing to find their efficacy range (data not shown), a 1:2 HTS solution in HEPES (Gibco, USA) and a 1:10 RP solution in Leibobitz’s L-15 medium (Sigma Aldrich, USA) were prepared. Culture medium was replaced by the preservation solutions and cell monolayers were immediately stored at 4°C. Controls at 4°C with unsupplemented Leibovitz’s L-15 medium (culture medium) and at 37°C (Control 37°C) with the same α-MEM formulation as before were also set up for each time point. An additional control comprising cells right before preservation was also set up (control 0h). After 3 and 7 days of storage, the preservation solutions were replaced with warm medium, and cells allowed to recover for 24 hours at 37°C (Fig 1).

### DNA quantification

At the end of the recovery phase, cells in the monolayers were lysed by osmotic and thermal shocks. A PicoGreen Quantification Kit (Invitrogen, United Kingdom) was used for double strand DNA (dsDNA) quantification. Samples were prepared according to manufacturer instructions and fluorescence was read in a Synergy HT microplate reader (Bio-Tek, USA).

### Flow cytometry

After each recovery period, cells were detached with TripLE Express (Thermo Scientific, USA), centrifuged and resuspended in cold PBS with 1% (v/v) FBS. Then, fluorescence-labeled mouse anti-human antibodies CD105-FITC (Bio-Rad, Netherlands), CD73-PE (BD Biosciences, Germany) and CD90-APC (BD Biosciences, Germany), were incubated 20 min at room temperature, using manufacturers recommended concentrations. Cells were washed with PBS, centrifuged, resuspended and analyzed in a FACSCalibur flow cytometer (BD Biosciences, USA). Cell viability was also assessed after labeling cells with 7-AAD (BioLegend, USA). Results were analyzed using Cyflogic software (1.2.1, CyFlo Ltd, Finland).

### Phalloidin staining

Cells were fixed in a buffered formalin solution, washed, and incubated with phalloidin (1:100) for 1h at room temperature. After repeated washes in PBS, cells were counterstained with DAPI (1:1000). Cells were then analyzed in a Zeiss Axio Observer inverted fluorescence microscope (Zeiss, Germany) and images acquired using ZEN 2 software (Zeiss, Germany).

### Immunocytochemistry

Cells in the monolayers were fixed in a buffered formalin solution. Non-specific binding was blocked with a 3% (w/v) BSA solution and cells were then incubated overnight at 4°C with primary antibodies rabbit anti-human laminin (1:30) (Abcam, United Kingdom), rabbit anti-human fibronectin (1:50) (Abcam, United Kingdom) and rabbit anti-human type I collagen (1:50) (Abcam, United Kingdom). After repeated washes in PBS, secondary antibody Alexa Fluor 488 donkey anti-rabbit (1:500) (Molecular probes, USA) was incubated with cells for 45 minutes at room temperature. Cells were washed in PBS and cell nuclei was counterstained with DAPI. Cell monolayers were then analyzed in a Zeiss Axio Observer inverted fluorescence microscope (Zeiss, Germany) and images acquired using ZEN 2 software (Zeiss, Germany).

### Alamar blue

The alamar blue assay is a widely known and used assay to determine cells’ metabolic activity [25]. Its active principle resazurin is non-toxic, cell permeant and an electron acceptor that changes color and becomes fluorescent when reduced by intracellular enzymes. It can therefore be used as an indicator of cellular metabolic activity. A working solution of alamar blue (Bio-Rad, Netherlands) was prepared in culture medium at a final concentration of 10% (v/v) and incubated with cell monolayers for 4h at 37°C and 5% CO_2_. After this time, the fluorescence was read in a Synergy HT microplate reader (Bio-Tek, USA).

### Western blot

For caspase 3 detection, cells were washed with ice cold PBS and incubated with lysis buffer composed of 50 mM of Tris-HCl Ph 7.0, 150 mM NaCl, 0.5% sodium deoxycholate, 0.1% (w/v) SDS, 1% of Triton x-100, protease inhibitor cocktail (1:100) and DTT (1:1000) (all from Sigma Aldrich, USA). The suspension was incubated on ice, centrifuged and the supernatant collected and frozen with liquid nitrogen. Total protein was quantified using a micro BCA protein assay kit (Thermo Scientific Pierce, USA), according to manufacturer instructions. In order to detect caspase 3, protein samples (30μg) were analyzed in a 16% SDS-PAGE gel. hASCs incubated for 24 hours in α-MEM supplemented with 10% FBS, 1% Antibiotic/Antimicotic and 100 μg/mL of mitomycin to induce apoptosis, were used as positive control while cells without mitomycin supplementation were used as negative control.

For laminin, fibronectin and type I collagen detection, cultures were washed with ice cold PBS, scraped from the bottom of the well, centrifuged and the resulting pellets were immediately frozen with liquid nitrogen and stored at -80°C until further analysis. For SDS-PAGE analysis, all samples were thawed and resuspended in 70ul of loading buffer. To detect fibronectin laminin and collagen type I, 15μl of all samples were run in a 8% SDS-PAGE gel. After transfer, the nitrocellulose membrane (Amersham Biosciences, USA), was blocked with 5% (w/v) skimed milk in TBS and incubated overnight at 4°C with the primary antibodies rabbit anti-human caspase 3 (1:700) (Cell Signaling Technology, USA), rabbit anti-human laminin (1:400) (abcam, United Kingdom), rabbit anti-human fibronectin (1:2000) (abcam, United Kingdom) and rabbit anti-human type I collagen (1:2000) (abcam, United Kingdom). After washing, the membrane was incubated with goat anti-rabbit antibody conjugated to alkaline phosphatase (1:5000) (Sigma Aldrich, USA). Finally, after washing, the membrane was incubated with NBT/BCIP color development kit (Bio-Rad, Netherlands), for signal visualization.

### Osteogenic and adipogenic differentiation assessment

After each time point has been reached and cells have recovered for 24 hours at 37°C, differentiation was initiated. For osteogenic differentiation, α-MEM was replaced with Dulbecco's Modified Eagle Medium (DMEM) (Sigma Aldrich, USA) supplemented with 10% FBS, 1% antibiotic/antimycotic, 10^−8^ M dexamethasone (Sigma Aldrich, USA), 50μg/ml ascorbic acid (Wako, USA) and 10 mM β- glycerophosphate (Sigma Aldrich, USA) and the medium was changed twice a week for 28 days. For adipogenic differentiation α-MEM was replaced with DMEM supplemented with 10% FBS, 1% antibiotic/antimycotic, 0.25 mM 3-Isobutyl-1-methylxanthine, 66 μm biotin, 34 μM D-pantothenic acid hemicalcium salt, 5 μM rosiglitazone, 1 μM Dexamethasone and 200 nM insulin (all from Sigma Aldrich, USA). The induction medium was changed twice a week for 10 days. After 10 days, the medium was replaced by maintenance medium (same composition of the induction medium except 3-Isobutyl-1-methylxanthine and rosiglitazone) for 18 days, twice a week. Adipogenic differentiation was confirmed after labelling the lipid droplets with Oil Red O solution. Osteogenic differentiation was confirmed after mineral deposition labelling with Alizarin red S solution. Images were acquired using a Zeiss Axiovert 40 inverted microscope (Zeiss, Germany).

For quantification of differentiation, images were processed using the software ImageJ (NIH Image Bethesda, MD) as described previously [26]. Shortly images were converted to a 24-bit RGB format, and the stained area quantified by selecting the pixels which presented higher than the considered threshold.

### Statistical analysis

Data is herein expressed as mean ± standard deviation of the results of DNA quantification, flow cytometry, metabolic activity and differentiation performed in triplicate for each of the 4 donors. Statistics were performed on GraphPad Prism 5. Shapiro-Wilk test for normality was applied and One-way ANOVA with Tukey’s *post-hoc* test used to assess differences between conditions. Significance levels were set to p < 0.05.

## Results

### Effect of hypothermic storage on hASC number, metabolic activity and marker expression

**Fig 2:**
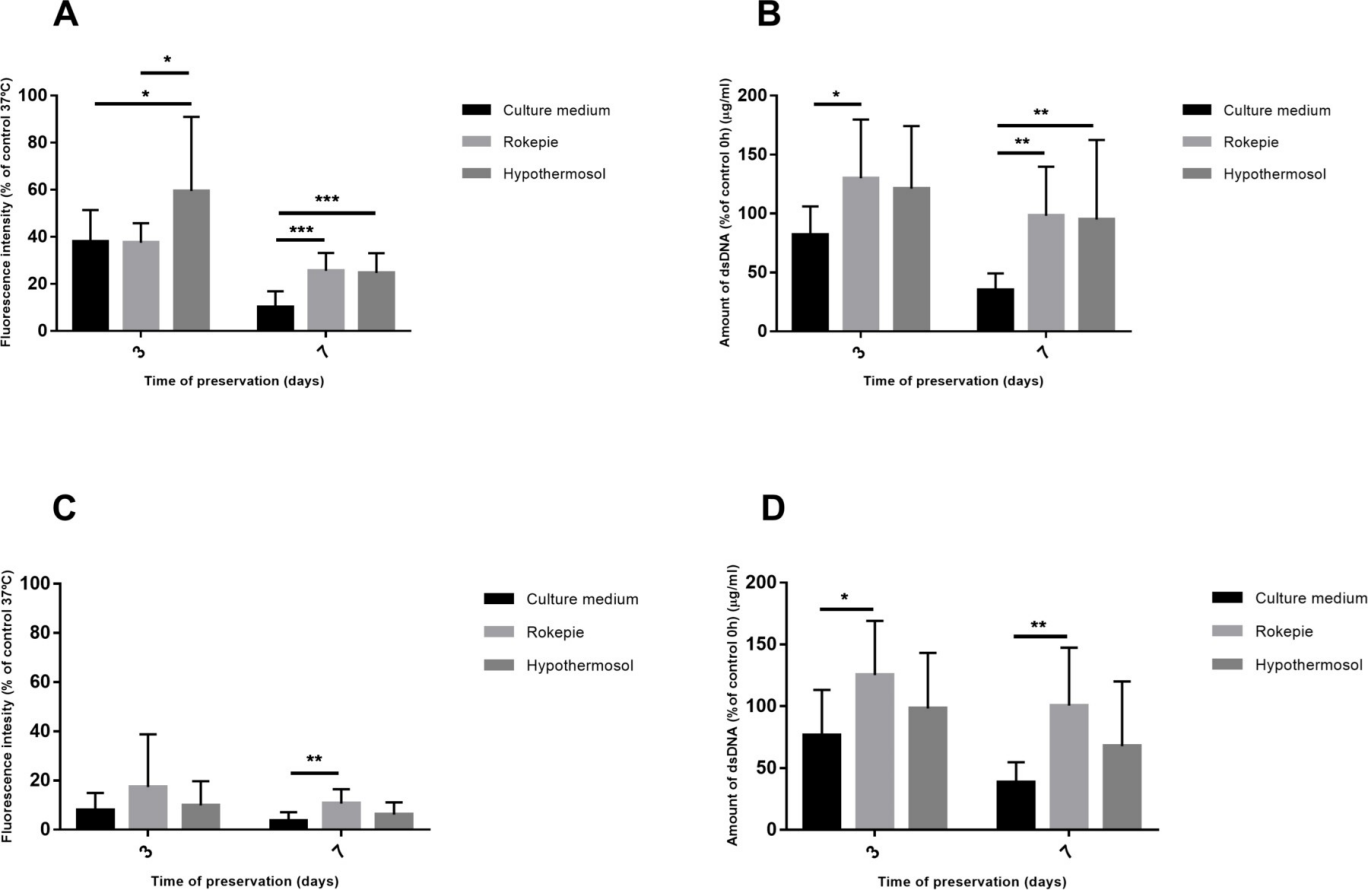
Metabolic activity and amount of dsDNA. Confluent (**A, B**) and non-confluent (**C, D**) hASCs were preserved at 4°C, in the presence and absence (Culture medium) of hypothermic storage solutions for 3 and 7 days. **A)** Metabolic activity of confluent cultures in the different conditions as given by fluorescence intensity after alamar blue assay. **B)** Amount of dsDNA in confluent hASCs cultured in the different conditions. **C)** Metabolic activity of non-confluent cultures in the different conditions as given by fluorescence intensity after alamar blue assay. **D)** Amount of dsDNA in non-confluent hASCs cultured in the different conditions. Metabolic activity and amount of dsDNA data are presented as mean±stddev of the % of the values for, respectively, the 37°C and 0h controls and were analyzed using one-way ANOVA and Tukey’s post-tests (*p < 0.05, **p < 0.01 and ***p < 0.001).

In order to assess the impact of hypothermic storage with RP and HTS over cell number and metabolic activity, cells were placed at 4°C for 3 and 7 days and allowed to recover for 24 hours at 37°C after each time point. All these treatments were compared with the control before preservation (control 0h). After 3 days of preservation, HTS allowed cells in the confluent monolayers to retain about 60% of the metabolic activity of the control 0h, significantly higher than for the RP and the unsupplemented groups, both presenting a metabolic activity around 37% of the 37°C control (Fig 2A). At day 7, both RP and HTS presented a metabolic activity of about 25% of the control 0h, significantly higher than the unsupplemented condition (10.16%) (Fig 2A). dsDNA quantification of confluent cultures revealed that when preservation solutions were used, cell content was maintained along the timepoints, while significant cell loss was found in the unsupplemented condition (Fig 2B). Previous reports suggest that the ECM might have a protective role to temperatures insults [27–30]. Therefore, non-confluent cultures (2.500 cells/cm2) were set up and subjected to the same conditions of the confluent cultures. In general, the metabolic activity of non-confluent samples as % of the respective control were much lower than the ones found for confluent cultures (Fig 2C). In terms of cell contents as given by dsDNA quantification, a trend similar to the one found in confluent cultures was verified (Fig 2D**).**

Changes in the expression of mesenchymal stem cell-associated surface markers after 4°C storage were screened by flow cytometry analysis. Before storage, close to 100% of cells exhibited positive expression of the markers CD73, CD90 and CD105 and no significant changes were observed after 3 and 7 days of storage for all the conditions (Table 1).

**Table 1:** Flow cytometry analysis of mesenchymal and viability markers. Flow cytometry analysis of confluent hASCs cultured at 4°C, in the presence and absence (culture medium) of hypothermic storage solutions for 3 and 7 days. A control culture at 37°C was also performed. Values for 0 hours correspond to cells before 4°C incubation. Mesenchymal markers CD105, CD90, CD73 as well as the viability marker 7-AAD were screened.

Flow cytometry					
	Condition	CD105	CD73	CD90	Dead Cells (7-AAD)
**0h**		99.12 ± 0.81	99.24 ± 0.86	99.33 ± 0.39	**1.25 ± 0.84**
**3days**	Control 37°C	97.58 ± 0.70	98.11 ± 1.42	99.90 ± 0.09	**2.55 ± 1.51**
	Culture medium	97.99 ± 1.72	98.80 ± 1.22	99.80 ± 0.10	**4.40 ± 1.58**
	Rokepie	99.83 ± 0.10	99.46 ± 0.63	100.00 ± 0.00	**3.26 ± 1.99**
	Hypothermosol	99.31 ± 0.52	99.07 ± 1.16	99.96 ± 0.06	**2.72 ± 1.57**
**7days**	Control 37°C	93.83 ± 5.19	98.42 ± 1.42	99.87 ± 0.14	**2.84 ± 2.34**
	Culture medium	94.51 ± 4.45	85.39 ± 26.75	99.83 ± 0.15	6.63 ± 1.90*
	Rokepie	99.43 ± 0.43	99.13 ± 0.88	99.90 ± 0.10	**4.70 ± 0.13**
	Hypothermosol	99.27 ± 0.74	98.73 ± 1.79	99.93± 0.09	**4.90 ± 1.34**

Flow cytometry data is presented as mean±stddev and was analyzed using one-way ANOVA and Tukey’s post-tests (*p < 0.05 in comparison to control at 37°C)

### Cell morphology, cytoskeleton organization and matrix integrity after storage

**Fig 3:**
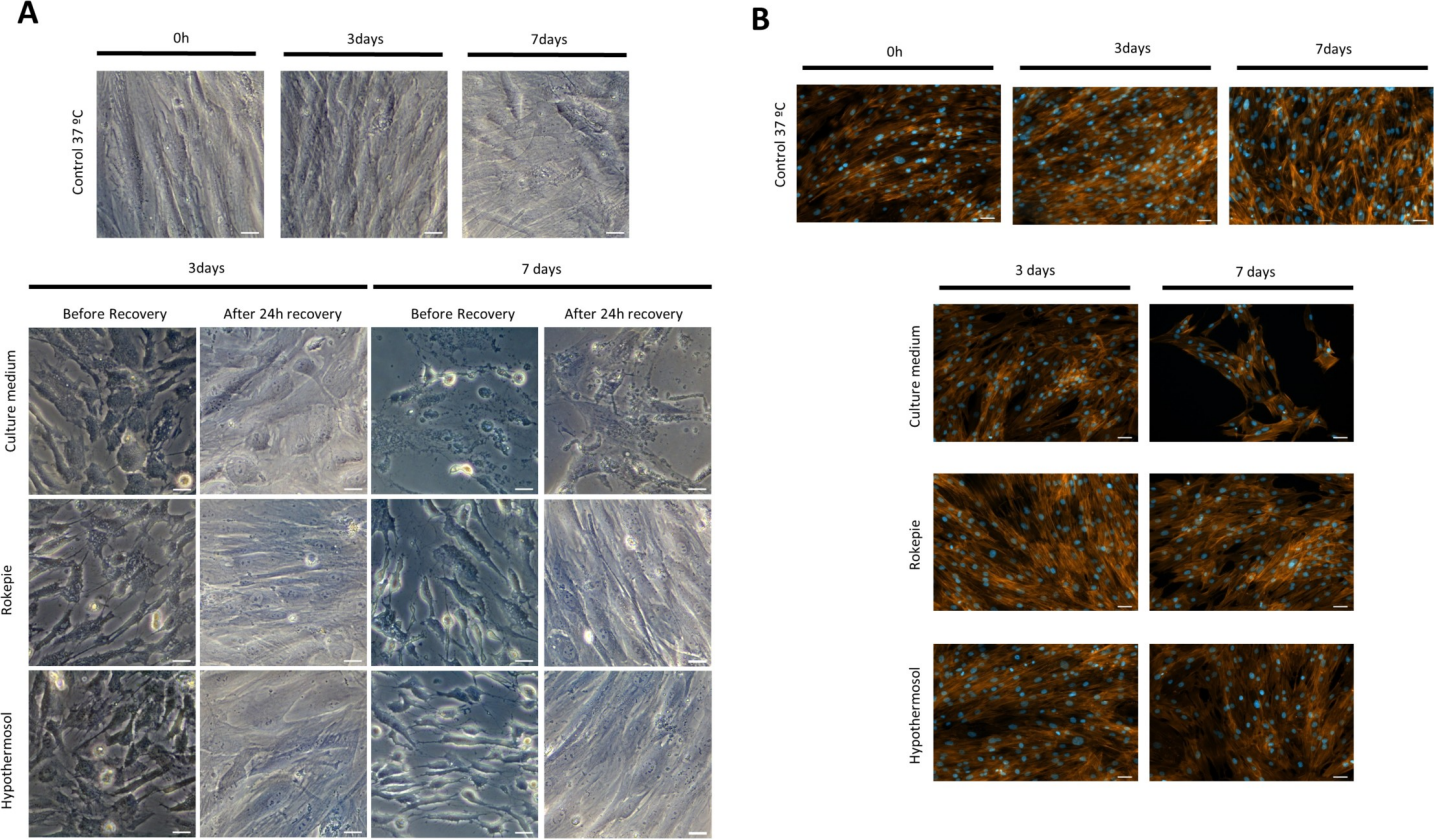
Brightfield and phalloidin staining images. Representative images of confluent hASCs preserved at 4°C, in the presence and absence (Culture medium) of hypothermic storage solutions for 3 and 7 days. Images of control culture at 37°C was also included. Values for 0 hours correspond to cells before 4°C incubation **A)** Morphology assessment at the end of preservation period, and after a respective recovery period of 24 hours. **B)** Phalloidin staining for the actin filaments (orange) performed after cultures being subjected to a recovery period of 24 hours. Cell nuclei were counterstained with DAPI (blue). Scale bar: 50μm.

Microscopy images were acquired for every condition after each time point and after the 24 hours recovery phase to evaluate cell morphology. Regardless of storage time, cells present a rounder shape immediately after being removed from the cold (Fig 3A). After a recovery phase of 24 hours, cells in the monolayers in the presence of the preservation solutions recovered their typical spindle-like shape to a morphology similar to cells in the control at 37°C. In the unsupplemented condition, this recovery was less pronounced and after 7 days of hypothermic incubation, cells without supplementation start to detach and disintegrate (Fig 3A). The significant level of cell detachment observed at day 7 for the unsupplemented group was further confirmed by phalloidin staining (Fig 3B).

**Fig 4:**
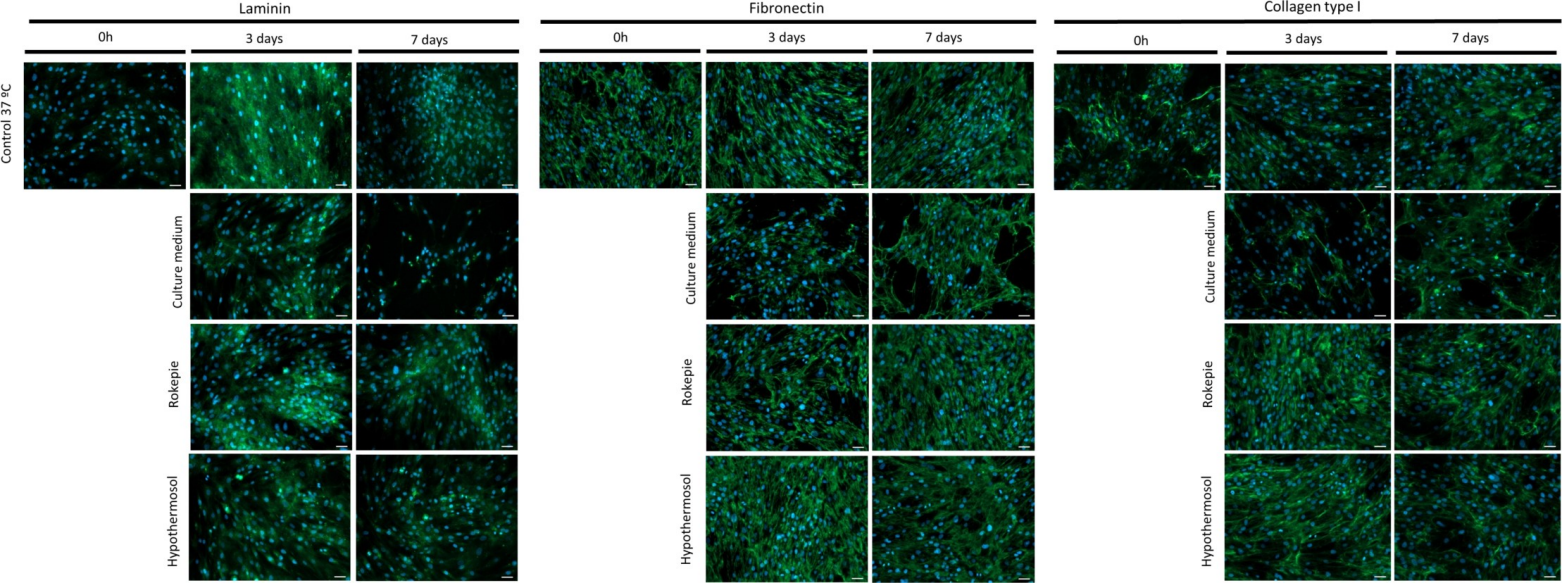
Immunocytochemistry images of expression of extracellular matrix proteins. Representative immunocytochemistry images of expression of extracellular matrix laminin, fibronectin and type I collagen (all in green) in confluent hASCs preserved at 4°C, in the presence and absence (Culture medium) of hypothermic storage solutions for 3 and 7 days. A control culture at 37°C was also included. Immunocytochemistry was performed after cultures were subjected to a respective recovery period of 24 hours. Values for 0 hours correspond to cells before 4°C incubation. Cell nuclei were counterstained with DAPI (blue). Scale bar: 50μm.

**Fig 5:**
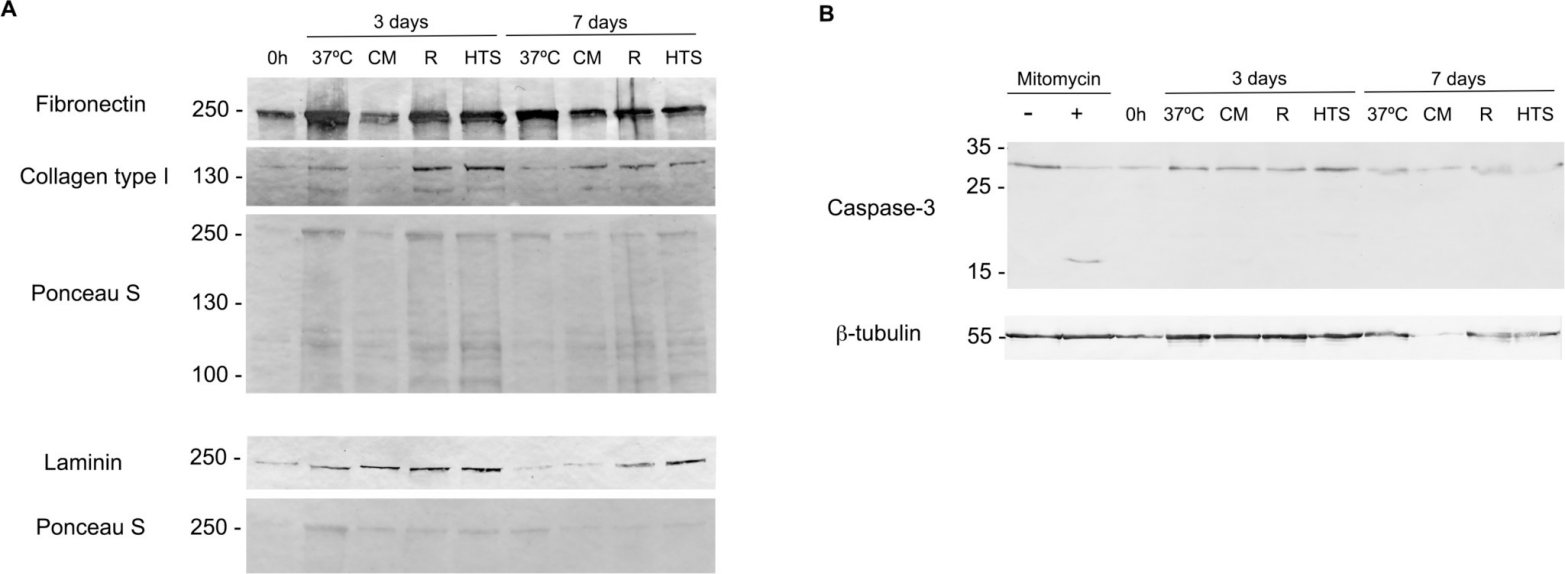
Western blot analysis of extracellular matrix and caspase 3 proteins. Western blot analysis of confluent hASCs preserved at 4°C, in the presence and absence (Culture medium) of hypothermic storage solutions for 3 and 7 days. A control culture at 37°C was also performed. The expression of extracellular matrix proteins fibronectin, type I collagen and laminin, as well as, caspase 3 activation expression was assessed. Samples were collected after cultures being subjected to a respective recovery period of 24 hours. For extracellular matrix proteins 15μl of each sample were loaded, whereas for caspase 3, 30μg were analyzed. Negative and positive controls for apoptosis, without and with mitomycin respectively, were also included to assess caspase activation. **A)** Ponceau S and **B)** β-Tubulin are shown as loading controls for fibronectin, type I collagen and laminin expression and caspase 3 activation, respectively.

Moreover, immunocytochemistry was used to qualitatively assess the expression pattern of ECM proteins laminin, fibronectin and type I collagen. After 3 and 7 days of preservation in both RP and HTS expression patterns remained similar to the control at 37°C. On the other hand, cells in the unsupplemented condition presented a clear change in the expression pattern of these ECM proteins, indicating disruption of ECM (Fig 4). ECM protein expression was then semi-quantitatively assessed by western blot. Similarly, to immunocytochemistry results, western blot results show that the expression of all of the assayed ECM proteins is severely affected by 4°C exposure and that RP and HTS can both mitigate this effect (Fig 5A).

### Assessment of necrosis and apoptosis: 7-AAD expression and caspase 3 activation

To assess if hypothermic storage was inducing cell death by necrosis or apoptosis, 7-AAD staining and caspase 3 activation were analyzed respectively by flow cytometry and western blot. Comparing to the 37°C control, no significant differences were observed in cell viability for the groups where preservation solutions were used. (Table 1). However, a reduction in cell viability was observed for the unsupplemented group although not reaching statistical significance after 3 days of storage. Caspase 3 activation was analyzed by SDS-PAGE and western blot. This protein (MW = 35 kDa) is present in healthy cells in its inactive form, being active only if cleaved (originating a 17 kDa and a 19 kDa fragment) when the apoptosis cascade is activated. Control cells cultured in the presence of mitomycin, an apoptosis inducer, displayed 2 bands of ~19 kDa and ~17 kDa corresponding to the active protein (Fig 5B). On the other hand, all the experimental conditions exhibit a unique band of ~35 kDa, suggesting caspase 3 non-activation.

### Differentiation potential of cells following storage

**Fig 6:**
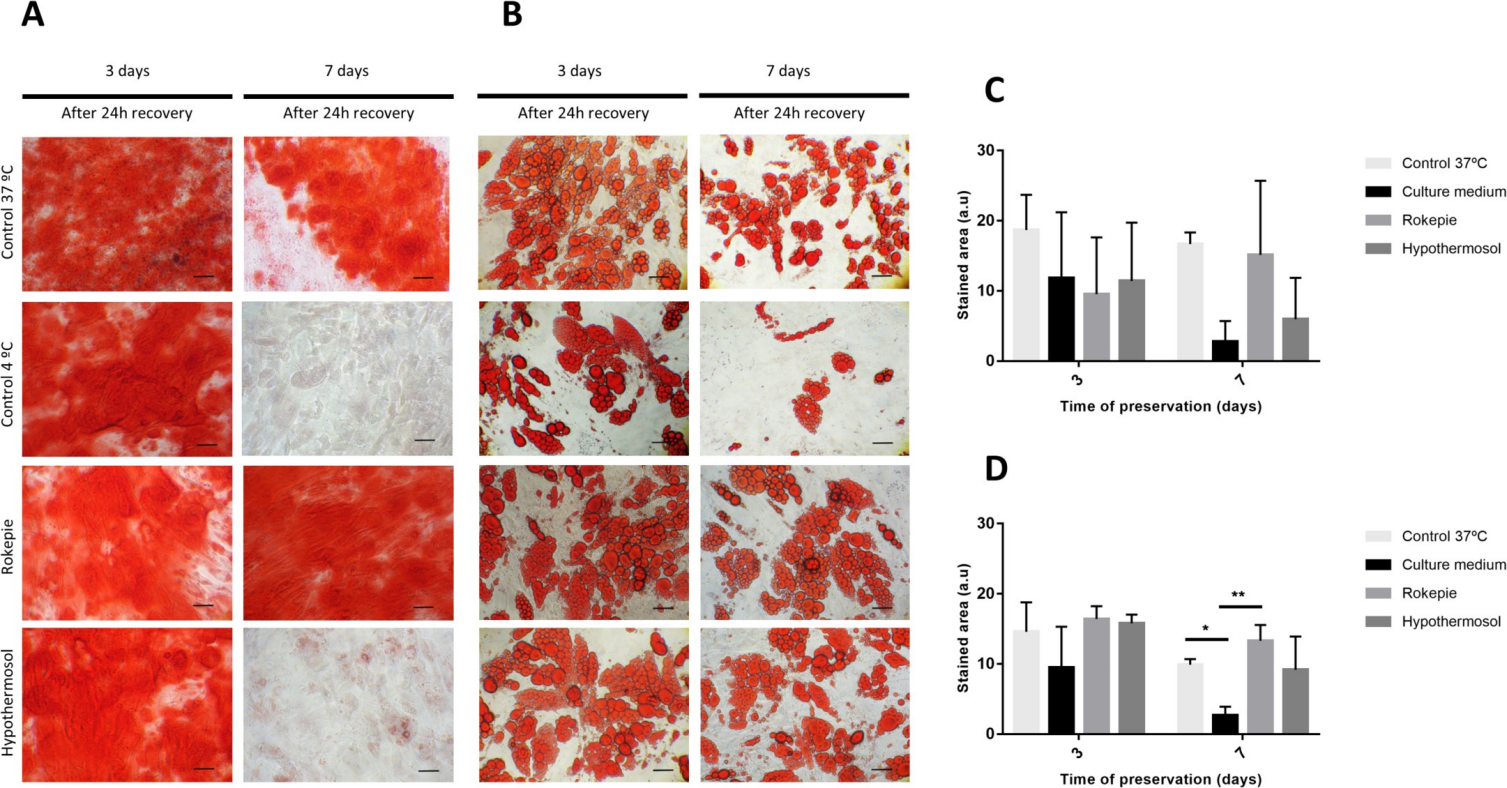
Differentiation potential and stained area quantification. Representative images of the differentiation potential and stained area quantification in confluent hASCs preserved at 4°C, in the presence and absence (Culture medium) of hypothermic storage solutions for 3 and 7 days. A control culture at 37°C was also included. **A)** Alizarin Red S staining for mineralization during osteogenic differentiation. **B)** Oil Red O staining for lipid accumulation during adipogenic differentiation. Scale bar: 100μm. **C)** Quantification of alizarin red S stained area by ImageJ software. **D)** Quantification of Oil red O stained area given by ImageJ. Stained area values are presented as mean±stddev and were analyzed using one-way ANOVA and Tukey’s post-tests (*p < 0.05 and **p < 0.01).

Differentiation was assessed using Alizarin Red S staining for mineralization (for osteogenesis) and Oil Red O staining for oil droplet deposition (for adipogenesis). Cell monolayers incubated at 4°C for 3 days presented osteogenic differentiation comparable with the 37°C control, both in the presence and absence of preservation solutions (Fig 6A). When storage time was increased to 7 days, only cell monolayers incubated with RP were able to maintain osteogenic differentiation potential similar to the 37°C control (Fig 6A). In the case of adipogenesis, hASCs monolayers incubated at 4°C for 3 and 7 days in the preservation solutions presented oil droplet deposition comparable to the control kept at 37°C (Fig 6B). However, adipogenic differentiation was weaker for cells in the unsupplemented condition, especially after 7 days of storage. Quantification of stained areas (Fig 6C and 6D) confirmed these trends, although no statistical differences were found in the case of the Alizarin Red staining.

## Discussion

Given the recent developments in the application of cell sheets for the regeneration of several tissues, a preservation strategy that ensures the maintenance of cell sheets viability and function as well as ECM properties would be a great step towards clinical application [31]. In preliminary studies in our lab, cryopreservation of cell sheets resulted in major ECM disruption (data not shown). Exposing cells to hypothermic temperatures in the presence of protective media or supplements, however, is a milder approach that prevents cell damage from ice nucleation and changes in solute concentration during cryopreservation. Based on this knowledge, we hypothesized that through the use of such media or supplements, is possible to temporarily store cell sheets at 4°C with reduced loss of viability and of matrix integrity.

Cell sheets can be produced using thermo-responsive dishes or retrieving sheets from normal culture surfaces by scraping. For both of these methodologies, the immediate step before retrieval is producing a confluent monolayer of cells where changes in cells before and after retrieval are mostly related with the cytoskeleton [32]. Considering this, in this study, cell sheet-like confluent cultures were used to test two different preservation strategies using either HTS or RP. In a first stage, metabolic activity and cell content were analyzed. HTS presented the best metabolic activity results after 3 days of storage probably due to the more complex and complete composition of this solution in comparison with RP. In addition to the ions that correct the cytoplasmic ionic imbalance in cells, HTS contains lactobionate that together with sucrose and mannitol are key components to counteract cell swelling [33] and dextran that acts as a colloid for oncotic support [34]. HTS had already been tested for hypothermic preservation of several cell types, maintaining cell viability in a range between 40% - 80% [24,35–37]. This variability of results makes evident that different cell types may react differently to the same preservation solution. hASCs in particular, have been preserved at 4°C with this storage medium with the help of alginate encapsulation but the percentage of viable recovery was only of 20% after 3 days of storage [28]. Our results, achieved without the generally detrimental encapsulation procedure, manage to be far better after the same preservation period (60%). On the other hand, cells preserved with RP fared less well with 37% of the activity measured for the 37°C control after 3 days of preservation. Unlike HTS, RP is a media supplement composed of the 6-chromanol derivative called SUL-109, known for its antioxidant capacity. According to literature, SUL-109 protective effects are due to the increase of mitochondrial complexes I and IV function [20] which will facilitate the transport of electrons in mitochondria and thus ATP production. Therefore, it is clear that the different mechanisms of action of HTS and RP may account for the superior performance of HTS. Interestingly, cells in non-confluent cultures, regardless of the presence or absence of preservation solutions, were shown to have much lower values of metabolic activity. This seems to confirm the protective role of ECM in the context of exposure to cold temperatures reported before [29,30].

Concerning cell content given by dsDNA analysis, both preservation solutions were able to maintain higher dsDNA levels than the unsupplemented group, where cell damage and loss is confirmed microscopically by higher degree of detached cells and cell debris. Hypothermic temperatures are known to slow the energy dependent processes, such as protein synthesis, ATP production and transport systems but cells continue to deplete nutrients and produce waste products. Decreased ATP production affects ion pumps in cell membrane leading to ionic imbalances, cytoskeleton disassembly and ultimately cell swelling [38] and rounding [39]. Preservation solutions seem to counteract these effects since in these groups, and contrary to the unsupplemented group, a full recovery of the typical spindle-shape morphology of hASCs after the 24-hour recovery phase was observed. The protective effect of the preservation solutions is further highlighted by significantly lower cell death in these groups vs the unsupplemented group as given by 7-AAD staining, which is also verified in non-confluent cultures (S1 Table). It was demonstrated before that the main causes for cell death during hypothermic preservation are cell lysis related with decrease in cell membrane stability (classical necrosis) [36,40–42] and caspase-mediated apoptosis [35–37,42–45]. Therefore, we analysed cell death using 7-AAD staining and caspase 3 (a main effector in the apoptotic process) activity. Despite the observed cell death, results show no activation of caspase 3 after the recovery phase in all conditions, which is highly suggestive of necrotic cell death. However, according to the current guidelines by the “Nomenclature Committee on Cell Death 2018” [46] to classify the type of cell death, what is considered as necrosis would be better described as accidental cell death whereas the caspase-related process would be considered regulated cell death. The latter, however, has many sub-routines, many not caspase dependent. With the analysis employed in this work, one cannot definitely say that all the cell death is due to necrosis since we have not screened for all the types of regulated cell death. An additional confounding factor is that while activation of regulated cell death could in fact be happening in response to the stress caused to cells by low temperatures, the amplification and execution of the pathway is energy dependent. Since cells under hypothermic temperatures are at a low energy production state, a switch in regulated cell death pathways can occur [23]. Therefore, and taking in account previous studies, a more correct interpretation of our data would be that cell death a) did not follow a caspase-dependent pathway, b) was most likely due to accidental cell death but c) other types of regulated cell death not involving caspase cannot be completely ruled out. Certainly, more in-depth studies to clarify the accidental vs regulated cell death issue must be employed.

In the particular case of cell sheets and their use in Tissue Engineering and Regenerative Medicine, ECM plays a key role since it acts as a totally biological scaffold that determine cell sheet-based constructs functionality [47]. Considering this, the impact of hypothermic exposure over ECM integrity and the protection given by preservation solutions were verified. RP and HTS were able to counteract a visible negative effect of hypothermia on ECM proteins expression confirmed by both immunocytochemistry and western blot. This deleterious effects seem to confirm the results of a previous study where oral mucosa cell sheets were successfully preserved with HBSS supplemented with ebselen during 7 days in hypothermia [48]. Taking in account the previous assertion that the ECM has a likely protective role on cells during exposure to hypothermic temperatures, it is reasonable to conclude that ECM protection is diminished by ECM damage. Therefore, the increased deleterious effects of hypothermia on cells seen from day 3 to day 7 of preservation seem to be, at least in part, the result of impaired ECM protection.

The use of preservation solutions was crucial to maintain hASC monolayers differentiation potential. While hypothermic preservation did not affect the expression of markers assessed during hASC characterization, which is in line with previous results with MSCs [23,28,37], functional assays for differentiation demonstrated an impact of hypothermia in their differentiation potential. RP and HTS were both able to rescue cells’ ability to differentiate into the adipogenic lineage to levels similar to the 37°C controls, but in the case of osteogenic differentiation, only RP was able to maintain these levels for the later time point. While it is clear that these cells can maintain at least a bi-potent differentiation capability, the intrapopulation heterogeneity is not herein addressed. This is a very important issue since some studies have shown that the composition of MSC populations is more complex at a biochemical level than previously thought and can be shaped by selective pressures imposed on cells by in vitro culture, impacting the composition, function, and therapeutic potency of those populations [49,50]. In fact, it is known that MSC populations are functionally heterogeneous, constituted by groups of cells with different differentiation capacities, ranging from tri-potent to bi-potent and uni-potent sub-populations [51–53]. Until now, how and to what extent do hypothermic temperatures affect each of those sub-populations is unknown since existing studies only assessed the capacity of polyclonal MSC populations to differentiate into 2–3 lineages [20,28,37,54]. With the methodology used in this study we cannot shed light on this issue since an analysis using single cell clones (CFU-F) would be needed. What we know is that the obtained results clearly suggest that hypothermic storage has different impacts on the differentiation potential of confluent hASC polyclonal populations, depending on the target lineages. But a clonal analysis of the different hASC sub-populations would be a valuable addition to reinforce the clinical significance of this study and will be pursued in future studies on the preservation of confluent cell cultures.

The ECM seems again to provide some degree of protection against hypothermia since testing in non-confluent hASC using the same conditions showed complete absence of osteogenic differentiation and decreased adipogenic differentiation (S1 Fig).

## Conclusions

With this work, it was demonstrated that HTS and RP were able to significantly preserve the metabolic activity and ECM integrity of cell sheet-like confluent hASC cultures during hypothermic storage for up to 7 days. This suggests both preservation solutions are excellent candidates for the design of efficient hypothermic preservation strategies for cell sheet-based regenerative medicine applications. Furthermore, while both preservation strategies were successful at protecting hASCs adipogenic potential, RP was the only one keeping hASCs osteogenic potential, which can be determinant for bone-related regeneration strategies.

## Supporting information

S1 Table Flow cytometry analysis of mesenchymal and viability markers. Flow cytometry analysis of non-confluent hASCs preserved at 4°C, in the presence and absence (Culture medium) of hypothermic storage solutions for 3 and 7 days. A control culture at 37°C was also included. Values for 0 hours correspond to cells before 4°C incubation. Mesenchymal markers CD105, CD90, CD73 screened as well as viability marker 7-AAD. Flow cytometry data presented as mean±stddev and was analyzed using one-way ANOVA and Tukey’s post-tests (*p < 0.05 and **p < 0.01 in comparison to control at 37°C).
(TIF)

S1 Fig Differentiation potential and stained area quantification. Representative images of the differentiation potential and stained area quantification in non-confluent hASCs preserved at 4°C, in the presence and absence (Culture medium) of hypothermic storage solutions for 3 and 7 days. A control culture at 37°C was also performed. **A)** Alizarin Red staining for mineralization during osteogenic differentiation. **B)** Oil Red O staining for lipid accumulation during adipogenic differentiation. Scale bar: 100μm. **C)** Quantification of alizarin red S stained area by ImageJ software. **D)** Quantification of Oil red O stained area given by ImageJ. Stained area values presented as mean±stddev and were analyzed using one-way ANOVA and Tukey’s post-tests (*p < 0.05).
(TIF)
