# Correction: Strategies for the hypothermic preservation of cell sheets of human adipose stem cells

**DOI:** 10.1371/journal.pone.0259406

**Published:** 2021-10-27

**Authors:** Sara Freitas-Ribeiro, Andreia Filipa Carvalho, Marina Costa, Mariana Teixeira Cerqueira, Alexandra Pinto Marques, Rui Luís Reis, Rogério Pedro Pirraco

The following information is missing from the Funding statement: This study was supported by ERC Project CapBed (grant number 805411).
