# Supplementary figures and images for: Strategies for the hypothermic preservation of cell sheets of human adipose stem cells
Source: PLoS One. 2019 Oct 15;14(10):e0222597. doi: 10.1371/journal.pone.0222597 (PMC6793945; doi:10.1371/journal.pone.0222597)

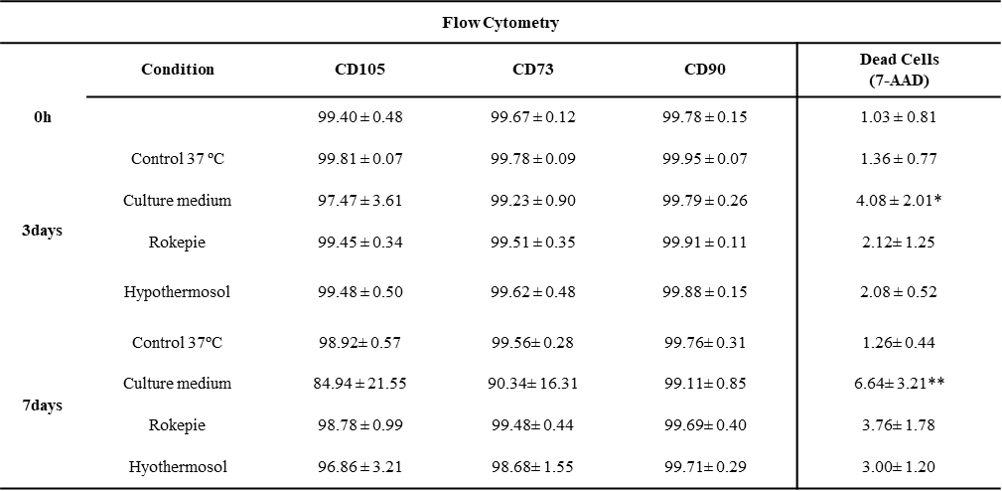

Supplement: S1 Table — Flow cytometry analysis of non-confluent hASCs preserved at 4°C, in the presence and absence (Culture medium) of hypothermic storage solutions for 3 and 7 days. A control culture at 37°C was also included. Values for 0 hours correspond to cells before 4°C incubation. Mesenchymal markers CD105, CD90, CD73 screened as well as viability marker 7-AAD. Flow cytometry data presented as mean±stddev and was analyzed using one-way ANOVA and Tukey’s post-tests (*p < 0.05 and **p < 0.01 in comparison to control at 37°C). (TIF) [file pone.0222597.s001.tif]

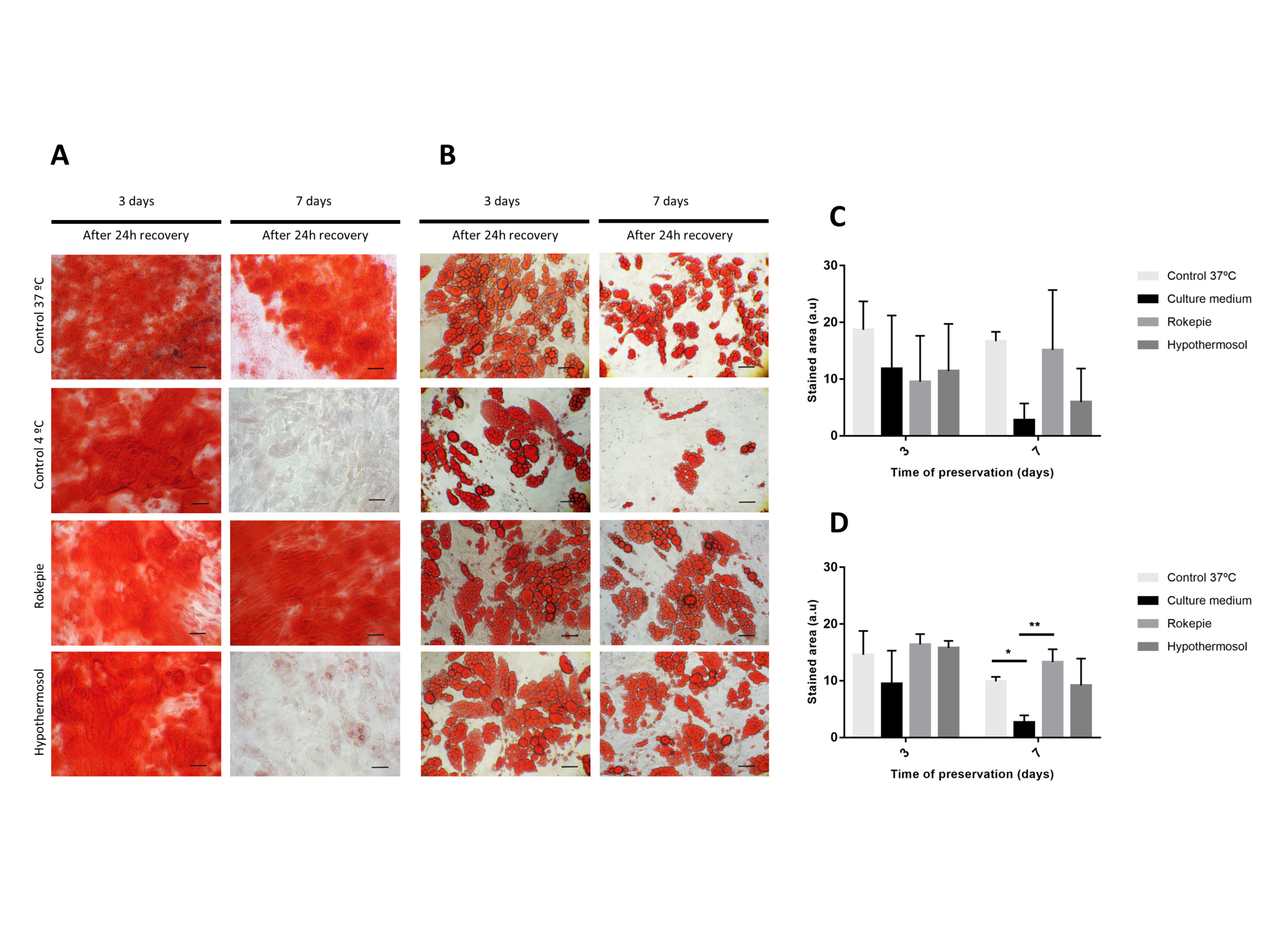

Supplement: S1 Fig — Representative images of the differentiation potential and stained area quantification in non-confluent hASCs preserved at 4°C, in the presence and absence (Culture medium) of hypothermic storage solutions for 3 and 7 days. A control culture at 37°C was also performed. A) Alizarin Red staining for mineralization during osteogenic differentiation. B) Oil Red O staining for lipid accumulation during adipogenic differentiation. Scale bar: 100μm. C) Quantification of alizarin red S stained area by ImageJ software. D) Quantification of Oil red O stained area given by ImageJ. Stained area values presented as mean±stddev and were analyzed using one-way ANOVA and Tukey’s post-tests (*p < 0.05). (TIF) [file pone.0222597.s002.tif]
